# The Dark Tetrad Traits Differentially Predict Gossip Frequency and Motives

**DOI:** 10.3390/bs16091556

**Published:** 2026-09-02

**Authors:** Adam C. Davis, Tracy Vaillancourt, Steven Arnocky

**Affiliations:** 1 Department of Social Sciences, Canadore College, North Bay, ON P1B 8K9, Canada; adam.davis@canadorecollege.ca; 2 Counselling Psychology, Faculty of Education, University of Ottawa, Ottawa, ON K1N 6N5, Canada; 3 Department of Psychology, Nipissing University, North Bay, ON P1B 8L7, Canada; stevena@nipissingu.ca

**Keywords:** personality, Dark Tetrad, Dark Triad, gossiping, motives

## Abstract

From an evolutionary perspective, gossip is argued to facilitate competition for survival and reproductive resources, such as status and mates. Previous work indicates that individual differences in the Dark Triad dimensions predict gossip motives. However, it is unclear how these “dark” traits might relate to gossip frequency, and the relations between the Dark Triad and motives for gossiping remain equivocal. Furthermore, researchers have yet to consider how everyday sadism as a part of the Dark Tetrad might predict gossip frequency and motives. In the current study, 516 North American adults (*M*_age_ = 37.90, *SD* = 12.76; 53.1% female) completed self-report measures for the Dark Tetrad, gossip frequency, and motives for gossiping. In a series of multiple regression analyses (controlling for age and biological sex), psychopathy emerged as the strongest positive predictor of gossiping, as well as the negative influence and protection motives. Machiavellianism positively predicted each gossip motive and was the only predictor of the information gathering and validation motive. These results extend the literature on personality and gossiping from an evolutionary viewpoint by helping to test novel links and clarify ambiguous associations between the Dark Tetrad dimensions and gossip frequency and motives influencing the behaviour.

## 1. Introduction

Far from idle, trivial chatter, gossip is central to the social lives of people across the globe for learning, solidifying friendships, courting mates, and gaining status, and as a form of indirect aggression (Baumeister et al., 2004; Dunbar, 2004). Gossip also likely functioned in a similar manner in ancestral contexts, underscoring the utility of taking an evolutionary perspective to understand the behaviour (Davis et al., 2019a; Ingram, 2014; McAndrew & Milenkovic, 2002; Reynolds et al., 2025). Gossiping involves the exchange of evaluative information (positive, neutral, or negative) about absent third-party others (Foster, 2004; Levin & Arluke, 1985; Testori et al., 2024; Wax et al., 2022). “Gossip” is not a single construct but includes how often people engage in the act (i.e., gossip frequency), associated attitudes and motives, contextual features (e.g., workplace gossip), content domains (e.g., physical appearance), valence (e.g., positive), and perceptual aspects (e.g., responses among receivers). Importantly, higher-order dimensions of personality (e.g., Extraversion) have been linked to a gossip frequency (Robbins & Karan, 2020) and motives underpinning the behaviour (Hartung & Renner, 2013). Researchers have further explored how inter-individual variability in “dark” (i.e., sinister) personality traits, specifically the Dark Triad dimensions (narcissism, Machiavellianism, and psychopathy), might predict specific gossip motives (Hartung et al., 2019; Lyons & Hughes, 2015; Uçan & Avci, 2023). However, to our knowledge, investigators have yet to examine the relations between the Dark Triad traits and gossip frequency. Moreover, there continues to be uncertainty regarding the links between the dimensions of the Dark Triad and gossip motives, and researchers have yet to examine how everyday sadism as part of the Dark Tetrad (Buckels et al., 2013; Paulhus et al., 2020) might relate to the frequency of, and motivations for, gossiping. Therefore, our goals with the current study were to assess potential novel links between the Dark Tetrad and gossip frequency, and to help clarify equivocal associations between the Dark Triad characteristics and gossip motives.

### 1.1. The Importance of Gossip in Modern and Ancestral Contexts

Although often understood as the petty sharing of negative information about others (Hartung & Renner, 2013), gossip serves a far more pivotal social function in contemporary society (Foster, 2004). As societies become more institutionalized and complex, gossip becomes an indispensable means of acquiring information when direct communication is not possible (Demerath & Korotayev, 2015). Gossip accounts for a considerable proportion of daily interpersonal communication (Dores Cruz et al., 2021b); it is a widespread phenomenon that is culturally and historically ubiquitous (Dunbar et al., 1997). These insights have prompted scholars to consider the evolutionary significance of this behaviour (Pan et al., 2024).

As group size and the complexity of social life increased, gossip likely became an indispensable means of gathering and exchanging information about others. Some have even considered gossip to be the impetus for the evolution of language (Dunbar, 2004). Gossip is also a powerful vehicle through which to acquire knowledge about the triumphs and failures of others via observational learning (Baumeister et al., 2004; Jolly & Chang, 2021). It further facilitates social comparisons, thereby allowing people to gauge their standing relative to peers to motivate competition for desired resources (Morgan et al., 2022; Suls, 1977). Moreover, it can promote the development and solidification of friendships (Ellwardt et al., 2012; Jolly & Chang, 2021), as well as communicate knowledge of social norms (Foster, 2004). Gossiping about norm-violating behaviour (e.g., cheating) can help police deviance, enforce rules, and build trust (Barkow, 1992; Dunbar, 1996; Imada et al., 2022; Peters et al., 2017). The social importance of gossip is further conveyed through people’s enjoyment of the activity; it has evident recreational value (Foster, 2004; Outlaw & Baer, 2024).

Gossip not only allows us to learn about others within a social milieu; it also functions as a mechanism for status enhancement (McAndrew et al., 2007; Sun et al., 2023). These insights, along with earlier theorizing, led to the proposal that derogatory gossip may be a key weapon used in the context of mate competition, particularly for women who tend to favour the use of indirect aggression against same-sex rivals (Campbell, 2004; Davis et al., 2019a; McAndrew, 2014; Reynolds et al., 2025; Vaillancourt, 2013). Indeed, Davis et al. (2018a) showed that adults higher in intrasexual competitiveness, denoting a stronger proclivity to compete with same-sex others over survival (e.g., status) and reproductive resources (e.g., mates), were more likely to gossip and to enjoy the activity.

### 1.2. Motivations for Gossiping

Building on the work of others (e.g., Foster, 2004), Beersma and Van Kleef (2012) proposed four social motives that appear to underpin gossiping: (1) information gathering and validation, (2) social enjoyment, (3) negative influence, and (4) group protection. Although often not considered gossip, many of our daily verbal exchanges about absent third-party others revolve around information gathering; we want to know about people in our social circles and what they are up to. This might be about news, celebrity drama, attending social events, health, friendship and family dynamics, work-related matters, and dating relationships (Foster, 2004). A powerful aspect of this motive is that it allows us to vet and validate information about people and our understandings of reality (Rosnow, 1977). For many, gossiping is also a stimulating and enjoyable activity; it can be driven more by amusement (Foster, 2004). The popularity of gossip across the globe highlights the importance of the social enjoyment motive. That is, gossiping is a rewarding activity linked to feelings of satisfaction and pleasure (Hartung & Renner, 2013; Outlaw & Baer, 2024).

When asked about gossip, people often assume that it is self-serving behaviour meant to damage the social standing and perceived integrity of others (Beersma & Van Kleef, 2012). This speaks to the salience of the negative influence motive, which involves gossiping to manipulate people in the hopes of gaining status and causing reputational harm to targets. This type of gossip is characteristic of injurious behaviour that is more circuitous, called indirect aggression (Archer & Coyne, 2005; Davis et al., 2019a; Eriksson et al., 2025; Ingram, 2014; Vaillancourt, 2005). The group protection motive involves the policing and enforcement of social norms to protect the people we gossip with. Often, we learn about norm violations via gossip, such as a classmate being reprimanded for cheating, co-workers getting in trouble for truancy, and community members being arrested for breaking the law. In this way, gossip can teach us about social norms and the consequences for violating expectations, customs, and laws (Dunbar, 2004; Imada et al., 2022; Noon & Delbridge, 1993). These diverse motives help to demonstrate that gossip can serve an array of social functions (Baumeister et al., 2004; Beersma & Van Kleef, 2012; Jolly & Chang, 2021). Importantly, motivations for gossiping (Hartung & Renner, 2013), gossip frequency (Robbins & Karan, 2020), and attitudes toward gossiping (Stiff, 2019) all vary alongside higher-order dimensions of personality.

### 1.3. Dark Personality Traits and Gossiping

Some investigators have considered the role of individual differences in “dark” personality traits on gossip. This research tends to center on a constellation of socially aversive dispositions called the Dark Triad, which includes narcissism, Machiavellianism, and psychopathy (Paulhus & Williams, 2002; Petrov et al., 2026). Narcissism embodies grandiosity (i.e., pompous superiority), a need for validation, and egocentricity, whereas Machiavellianism denotes cynicism, manipulativeness, and exploiting others for one’s own gain. Psychopathy centers on impulsiveness, callousness, and antisociality. These three dark traits are distinct dimensions of personality, but they share an overlapping core captured by low HEXACO Honesty–Humility; interpersonal manipulation, rule-breaking, material greed, and inflated self-importance constitute the fabric that stitches together these malevolent characteristics (Book et al., 2015). Evolutionary scholars have further theorized that the Dark Triad may serve an adaptive function in promoting a short-term mating strategy (Carter et al., 2014; Davis et al., 2019b; Jonason et al., 2009). Since the Dark Triad traits positively predict indirect aggression (Eriksson et al., 2025), which includes negative gossip, social exclusion, and rumour spreading, it is prudent to more closely examine the links between these sinister dimensions with various aspects of gossip, including gossip frequency, attitudes toward gossiping, and gossip motives. In this growing literature, researchers have used different methodologies to study the Dark Triad traits and gossip (see Table 1).

In an online sample of adults, Stiff (2019) found that Machiavellianism positively predicted favourable attitudes toward gossiping. Across five studies, Hales et al. (2025) found that American adults higher in narcissism were more open to being hypothetically gossiped about by others across scenarios that varied in valence (positive, negative, ambiguous, and no gossip).

Wantante (2024) studied the links between employees’ perceptions of a leader’s Dark Triad traits and the likelihood of sharing positive and negative workplace gossip among Ugandan non-governmental organizations. They found that when leaders were perceived to have higher levels of narcissism, Machiavellianism, and psychopathy, this encouraged defamatory gossip among subordinates toward supervisors.

In terms of motives to gossip, among adults in the United Kingdom, Lyons and Hughes (2015) found that negative influence was positively correlated with each Dark Triad trait, whereas none of these dimensions correlated with information gathering and validation. Primary psychopathy (i.e., callousness) and narcissism correlated positively with social enjoyment motives, whereas secondary psychopathy (i.e., impulsivity) and narcissism correlated positively with group protection. Despite diverse motives underpinning gossip for the Dark Triad, as anticipated, relations between the Dark Triad traits and negative influence were the strongest.

Hartung et al. (2019) examined the links between the Dark Triad and gossip motives among German adults. They found that the “motivational pattern” was similar across private and workplace contexts. Information validation and information gathering were measured as separate motives. Another salient motive that was studied was relationship building. Multiple regression analyses for gossip in a workplace context revealed that narcissism positively predicted information gathering, information validation, group protection, relationship building, and social enjoyment motives, whereas Machiavellianism positively predicted negative influence and information gathering. In a private context, Machiavellianism positively predicted information gathering, whereas narcissism predicted information validation, relationship building, and negative influence. Surprisingly, psychopathy did not predict any motive in either context. However, psychopathy did positively correlate with negative influence across contexts.

Uçan and Avci (2023) examined the cross-sectional links between organizational gossip and the Dark Triad traits among secondary teachers and university academic staff in Turkey. Across both samples, each Dark Triad trait correlated positively with organizational gossip for the purpose of information gathering and relationship building. Machiavellianism and psychopathy both correlated negatively with the perception that gossip produces organizational harm. Furthermore, the Dark Triad traits mediated the positive links between abusive supervision, whereby supervisors were perceived to humiliate, derogate, and withhold important information from employees, and information-gathering and relationship-building gossip.

Buckels et al. (2013) expanded upon the Dark Triad to include the fourth sinister disposition of everyday sadism, describing enjoyment and pleasure from the humiliation and suffering of others. Everyday sadists are callous, like those higher in psychopathy; however, they do not share the same inclination for impulsivity or criminality (Paulhus, 2014; Ward, 2025). Enjoyment of cruelty in socially sanctioned ways (e.g., consuming violent media, experiencing schadenfreude, and perpetrating internet trolling) is central to sadism, not grandiosity (narcissism) or manipulation (Machiavellianism). Thus, sadism shares important conceptual and empirical overlap with the Dark Triad traits, but it is a distinct construct that accounts for unique variability in predicting antisocial behaviour (e.g., aggression; Buckels et al., 2019). Researchers have not yet examined how everyday sadism as a part of this expanded Dark Tetrad model might relate to gossip frequency and motives. However, like the members of the Dark Triad (Eriksson et al., 2025), everyday sadism has been shown to positively predict the use of indirect aggression (Davis & Vaillancourt, 2023), which includes derogatory gossip.

### 1.4. The Influence of Age and Sex on Gossip

Previous work suggests that gossip frequency declines across the lifespan (Massar et al., 2012), particularly for negative gossip (Robbins & Karan, 2020). This makes sense given that individuals compete more intensely during adolescence and emerging adulthood for mates and status using indirect modes of aggression like gossip (Davis et al., 2019a; Fernandez et al., 2014; Vaillancourt, 2013). Sex and gender differences have also been found (Davis et al., 2018b). Women appear to gossip more than men (Litman & Pezzo, 2005; Robbins & Karan, 2020), particularly about physical appearance and social information (Davis et al., 2018a; Levin & Arluke, 1985; Nevo et al., 1993; Watson, 2012). Compared to men, women are more likely to express concern motivations for gossiping, which allows one to avoid being stigmatized and labelled as an untrustworthy and/or malicious gossiper (Reynolds et al., 2025). Despite these sex differences, mating reputation gossip has been found to be sex-invariant (De Backer et al., 2007). Furthermore, men and women who are intrasexually competitive appear to use gossip to the same extent (Davis et al., 2018a). And the links between the Dark Triad and gossip motives have not been found to vary significantly by sex (Hartung et al., 2019; Uçan & Avci, 2023).

### 1.5. Present Study

To our knowledge, researchers have not examined whether those higher in “dark” dispositions gossip to a greater extent, that is, have a higher frequency of gossiping in general. Higher levels of narcissism and primary psychopathy (Lyons & Hughes, 2015) are related to the social enjoyment gossip motive, and elevated Machiavellianism is linked to favourable attitudes toward gossiping (Stiff, 2019). A stronger expression of everyday sadism predicts the use of indirectly aggressive behaviour, such as social exclusion and derogatory gossip (Davis & Vaillancourt, 2023). Thus, we expected that the Dark Tetrad traits would positively predict gossip frequency (Hypothesis 1).

More research is needed to clarify the equivocal links between the Dark Triad and gossip motives, and researchers have yet to study everyday sadism in this dynamic. The Dark Triad dimensions have been positively linked to the negative influence gossip motive (Hartung et al., 2019; Lyons & Hughes, 2015), and the Dark Tetrad traits all positively predict indirect aggression (Davis & Vaillancourt, 2023). Narcissism and psychopathy appear to be positively associated with the social enjoyment gossip motive, whereas the relations with Machiavellianism have been less consistent (Lyons & Hughes, 2015; Hartung et al., 2019). Nonetheless, endorsement of gossip is positively linked with Machiavellianism (Stiff, 2019), suggesting greater enjoyment in the activity. Like psychopathy, everyday sadism may be related to enjoyment of gossiping to harm others via malicious gossip. Therefore, the Dark Tetrad traits were all expected to positively predict the negative influence and social enjoyment gossip motives (Hypothesis 2).

Positive links between narcissism and Machiavellianism and gossiping for information gathering and validation have been reported (Hartung et al., 2019; Uçan & Avci, 2023). The relation between psychopathy with these motives is unclear. Those higher in psychopathy and sadism are particularly antisocial (Buckels et al., 2019), and psychopathy is related to emotional indifference (Shukla & Upadhyay, 2025). By contrast, the search for validation encourages narcissists to obtain social feedback (Ohu & Jones, 2025). Machiavellians are distrustful and strive to extract social information to manipulate others (Bereczkei, 2018). Thus, narcissism and Machiavellianism were expected to positively predict the information gathering and validation gossip motive, whereas psychopathy and sadism were not (Hypothesis 3).

The group protection motive has been reliably associated with narcissism (Hartung et al., 2019; Lyons & Hughes, 2015). However, its links to psychopathy and Machiavellianism are equivocal. Despite notable deficits in empathy (Shukla & Upadhyay, 2025), in-group concerns can compel those higher in psychopathy toward protecting close friends, allies, and family (Arbuckle & Cunningham, 2012). The same cannot be said for everyday sadism. To successfully influence individuals, particularly in a workplace context, those higher in Machiavellianism may strategically protect social relationships until they have outlived their usefulness (Hartung et al., 2019). Nonetheless, Machiavellians are cynical and unlikely to help others at a cost to themselves (i.e., altruism; Trahair et al., 2022). Consequently, narcissism and psychopathy may positively predict the group protection gossip motive, but not Machiavellianism and sadism (Hypothesis 4).

## 2. Materials and Methods

### 2.1. Participants

For the current study, 564 North American adults agreed to participate in the “Status, Personality, and Aggression Study” hosted via Amazon Mechanical Turk (MTurk) to complete an online SurveyMonkey questionnaire. Data for 31 participants were removed for failing to enter a unique code at the end of the survey, and data for 13 individuals were removed for duplicate survey submissions (only the first survey responses were retained). Two participants fell outside of the eligible age range (18–65 years) for the study, which was guided by research showing that gossiping (Massar et al., 2012) and dark traits (Klimstra et al., 2020) decline across the lifespan. Data for two participants were further excluded because they failed the validity check. Therefore, the final analytic sample included 516 adults (*M*_age_ = 37.90; *SD* = 12.76; range = 18–61), 274 (53.1%) of whom indicated that their assigned sex at birth was female. Of the sample, 416 (80.6%) selected “White” as their ethnic/racial background, and 175 (34.1%) adults selected “completed undergraduate degree” for their educational level.

### 2.2. Measures

#### 2.2.1. Dark Personality Traits

To measure individual differences in the Dark Tetrad, the 28-item Short Dark Tetrad (SD4; Paulhus et al., 2020) was used. This measure includes four subscales: narcissism (e.g., “I know that I am special because people keep telling me so”), Machiavellianism (e.g., “It’s not wise to let people know about your secrets”), psychopathy (e.g., “People often say I’m out of control”), and sadism (e.g., “Some people deserve to suffer”). Responses to items were recorded with a 5-point Likert-type scale ranging from 1 (Strongly disagree) to 5 (Strongly agree). Items for each subscale were averaged to calculate mean scale scores. Higher scores denoted a greater expression of dark personality traits. The Narcissism (α = 0.88), Machiavellianism (α = 0.80), Psychopathy (α = 0.90), and Sadism subscales (α = 0.86) were all internally consistent.

#### 2.2.2. Gossip Frequency

Following Nevo et al. (1993) and their Tendency to Gossip Questionnaire, to measure individual differences in the proclivity to gossip, the 13-item Gossip Frequency Scale was created in the current study (see Supplemental Materials for a complete list of items). Given varying understandings of gossip (Dores Cruz et al., 2021a), participants first read a definition of gossip: Gossip involves sharing information with other people about someone who is not currently present. Gossip can be positive (e.g., talking about someone else’s achievements), negative (e.g., making fun of someone behind their back), or neutral. Gossip can take place in a face-to-face conversation, on the phone, through email, text messaging, or on social media. Using a 5-point Likert-type scale ranging from 1 (Never) to 5 (Always), participants were asked to report how often they gossiped about achievement at school and work, physical appearance, family, friendships, and romantic relationships, and rivals, as well as celebrities (e.g., actresses, professional athletes, politicians, YouTube personalities, etc.). Items were averaged to create a total mean scale score, with higher scores describing a greater tendency to gossip. The Gossip Frequency Scale was found to be internally consistent (α = 0.92) in the present study.

#### 2.2.3. Gossip Motives

To measure varying motivations for gossiping, the 22-item Motives to Gossip Questionnaire created by Beersma and Van Kleef (2012) was used. This measure has four subscales relating to distinct motives for gossiping: Information Gathering and Validation (e.g., “To find out whether the person I was talking with agreed with me”), Social Enjoyment (e.g., “To have a good time”), Negative Influence (e.g., “To damage the reputation of the person we talked about”), and Group Protection (e.g., “To protect the person I was talking with against the person we were talking about”). Participants responded to items using a 7-point Likert type scale, ranging from 1 (Completely disagree) to 7 (Completely agree). Scores for each subscale were averaged to create mean scale scores, with higher scores describing stronger motivations for gossiping. The Information Gathering and Validation (α = 0.96), Social Enjoyment (α = 0.92), Negative Influence (α = 0.91), and Group Protection (α = 0.86) subscales were internally consistent in the current study.

#### 2.2.4. Validity Check

Following the Conscientious Responder Scale (Marjanovic et al., 2014), five items were used to determine non-purposeful responding (e.g., “To answer this question, please choose option number four, ‘Very likely’”). The items were imbedded in randomly selected scales in the online survey. Participants who failed to correctly respond to three or more items were flagged for non-purposeful responding.

### 2.3. Procedure

A “human intelligence task” (i.e., HIT) containing a description of the research was used to invite individuals on MTurk to participate in the “Status, Personality, and Aggression Study.” Eligibility criteria included: (1) adult between the ages of 18–65, (2) currently residing in Canada or the United States, and (3) having a lifetime HIT approval rating of ≥80 (i.e., number of assignments approved divided by number of assignments ever completed). Interested participants were rerouted to SurveyMonkey to complete an online questionnaire containing randomly ordered self-report measures of interest. Participants were compensated with $5.00CAD if they completed the entire survey and passed the validity check. The current research was approved by an appointed institutional ethics committee from the University of Ottawa. Data collection for the current study occurred from May to June in 2021, prior to the widespread use of generative artificial intelligence software (e.g., ChatGPT) that can undermine the validity of crowdsourced online data (Asher et al., 2026).

## 3. Results

SPSS (version 31) was used to conduct all analyses in the present study. Descriptive statistics were calculated for all measured variables (see Table 2). Skewness and kurtosis values fell within acceptable ranges (skewness < +/−3 and kurtosis < +/−10; Kline, 2016). Independent-samples *t*-tests indicated that sadism differed significantly between the sexes—*t*(512) = −5.37, *p* < 0.001, *d* = 0.48—with men (*M* = 2.79, *SD* = 0.90) scoring higher than women (*M* = 2.33, *SD* = 1.03). Compared to men (*M* = 2.41, *SD* = 0.80), women (*M* = 2.61, *SD* = 0.83) also reported a greater frequency of gossiping, with *t*(512) = 2.75, *p* = 0.006, *d* = 0.25. No other statistically significant sex differences were found. Bivariate correlations indicated that age correlated negatively with narcissism (*r* = −0.17, *p* < 0.001), Machiavellianism (*r* = −0.25, *p* < 0.001), psychopathy (*r* = −0.28, *p* < 0.001), and sadism (*r* = −0.33, *p* < 0.001). Age also shared negative relations with gossip frequency (*r* = −0.19, *p* < 0.001), as well as the social enjoyment (*r* = −0.15, *p* < 0.001) and negative influence (*r* = −0.20, *p* < 0.001) gossip motives.

A series of bivariate correlations were then conducted to examine the associations between measured constructs of interest (see Table 2). Of note, each gossip motive correlated positively with the Gossip Frequency Scale, supporting its convergent validity. Each Dark Tetrad dimension also correlated positively with gossip frequency, in addition to the four motives for gossiping.

To test Hypothesis 1, a multiple regression analysis was conducted. Gossip frequency was entered as the dependent variable, age and biological sex (female = 1, male = 2)^1^ were entered at the first step to control for their influence, and the Dark Tetrad traits served as the predictors at the second step. Overall, the model (including the covariates) was significant, *F*(6, 507) = 90.70, *p* < 0.001, *R*^2^ = 0.52. At the first step, age (*b* = −0.13, β = −0.21, *p* < 0.001) and sex (*b* = −0.25, β = −0.15, *p* < 0.001) negatively predicted gossip frequency. At the second step, narcissism (*b* = 0.16, β = 0.19, *p* < 0.001), Machiavellianism (*b* = 0.12, β = 0.11, *p* = 0.004), psychopathy (*b* = 0.28, β = 0.35, *p* < 0.001), and sadism (*b* = 0.17, β = 0.21, *p* < 0.001) all positively predicted gossip frequency. The tolerance values (0.36–0.70) and variable inflation factors (VIFs; 1.44–2.81) for the Dark Tetrad traits fell within an acceptable range, indicating no concerns with multicollinearity (Kim, 2019).

Four multiple regression analyses were conducted to test Hypotheses 2–4. For each model, the four gossip motives were entered as dependent variables, age and sex were entered at the first step as covariates, and the four Dark Tetrad traits were entered at the second step as independent variables. In the first model (see Table 3 for results), information gathering and validation was the outcome. Overall, the model (including the covariates) was significant, *F*(6, 507) = 13.22, *p* < 0.001. Controlling for the shared variance among the Dark Tetrad traits, only Machiavellianism positively predicted this gossip motive. The second model, with social enjoyment entered as the dependent variable, was significant, *F*(6, 507) = 16.53, *p* < 0.001. Age negatively predicted the social enjoyment motive at the first step, whereas narcissism and Machiavellianism positively predicted this motive at the second step of the model.

For the third model (see Table 4 for results), negative influence was entered as the criterion, and the overall model was significant, *F*(6, 507) = 42.74, *p* < 0.001. At the first step, age negatively predicted the negative influence gossip motive, and Machiavellianism, psychopathy, and sadism positively predicted this outcome. For the last model, group protection was the outcome, which was also significant, *F*(6, 507) = 16.50, *p* < 0.001. Neither age nor sex predicted this motive at the first step. Machiavellianism and psychopathy were found to positively predict the group protection gossip motive.

### Exploratory Suppression and Redundancy Analyses

Narcissism correlated positively with the negative influence gossip motive with a medium effect size (*r* = 0.34; Gignac & Szodorai, 2016) but had a non-significant negative relation in the multiple regression model (β = −0.08). This signaled a potential suppression effect, where adding variables to a model significantly changes the strength and/or direction of an original predictor and outcome (Paulhus et al., 2004). Psychopathy has previously been shown to have a suppressing effect on narcissism (Davis et al., 2019b), and as such, we examined whether something similar was happening in the multiple regression model. Controlling for age and sex at Step 1, narcissism positively predicted negative influence when entered alone at Step 2 (see Table 5). Adding psychopathy at Step 3 resulted in a significant change in *R*^2^ and rendered narcissism a non-significant predictor. The Sobel test (MacKinnon et al., 2000) supported a significant suppression effect for psychopathy on narcissism in predicting negative influence, *z* = 5.81, *p* < 0.00. In contrast, psychopathy’s relationship with negative influence did not change when narcissism was added to the model, and the Sobel test indicated that the influence of narcissism on psychopathy in the regression model was non-significant, *z* = −0.12, *p* = 0.901.

At the bivariate level, sadism was positively correlated with gossip for group protection (*r* = 0.28), which became non-significant in the multiple regression model. Some have argued (e.g., Jonason et al., 2017) that sadism may be redundant with psychopathy in predicting certain criterion variables, because of their shared conceptual overlap. If so, when predicting the group protection gossip motive, the beta coefficient for sadism should be reduced when psychopathy is added to the model, but there should not be a significant change in *R*^2^ (Paulhus et al., 2004). Similarly, adding sadism to a model should reduce psychopathy’s beta coefficient in predicting group protection with no significant change in *R*^2^. Controlling for age and sex at Step 1, sadism positively predicted group protection at Step 2 of the model. Adding psychopathy to the model rendered sadism a non-significant predictor of the outcome variable. However, there was a significant change in *R*^2^. In contrast, adding sadism to a model with psychopathy reduced its beta coefficient, and there was no significant change in *R*^2^. Thus, there was insufficient evidence to support a redundancy effect between psychopathy and sadism.

## 4. Discussion

Gossip comprises a significant proportion of our day-to-day verbal communication as human beings, which often has a neutral or positive valence about absent third-party others (Dores Cruz et al., 2021b; Robbins & Karan, 2020). It is a cross-culturally and historically ubiquitous phenomenon that likely helped solve adaptive problems related to survival and reproduction, underscoring the utility of an evolutionary perspective in studying the behaviour (Dunbar et al., 1997; Hess & Hagen, 2021; Ingram, 2014; McAndrew & Milenkovic, 2002; Pan et al., 2024). Despite these universal features, individual differences impact the frequency of (Davis et al., 2019a) and motivations for gossiping (Hartung & Renner, 2013). Some investigators have also examined how inter-individual variability in “dark” (i.e., sinister) dispositions relate to gossip motives (Hartung et al., 2019; Lyons & Hughes, 2015; Uçan & Avci, 2023; Wantante, 2024).

In support of Hypothesis 1, when providing North American adults with a definition of gossip (positive, neutral, negative) and asking them to report how often they gossiped about various topics (e.g., physical appearance, friends, rivals, politicians, athletes, etc.), each Dark Tetrad trait positively predicted gossip frequency. Gossip has a salient competitive function in helping to vie for valued social (e.g., status) and reproductive resources (e.g., mates; McAndrew, 2014). Negative gossip can be used as a mode of indirect aggression to compete for popularity, dominance, friends, and relationship partners (Davis et al., 2019a; Ingram, 2014; Vaillancourt & Krems, 2018). Even in the name of cooperation, gossip is used competitively to appear more generous, build trust, and form alliances (Giardini et al., 2021). Competitive contexts, wherein resources are scarcer and more valuable, also promote gossiping, particularly derogatory gossip (Hess & Hagen, 2021). Moreover, young adults who are more competitive toward same-sex rivals (i.e., intrasexual competitiveness) gossip more often and show greater enjoyment of the activity (Davis et al., 2018a). Similarly, the Dark Tetrad traits are positively associated with competitiveness (Furnham & Cuppello, 2024), indirect aggression, and striving for dominance status via coercion and intimidation (Davis & Vaillancourt, 2023). Thus, it is intuitive that these more sinister personality traits would predict a greater frequency of gossip. A comprehensive understanding of gossip frequency requires examining motives that promote this behaviour (Beersma & Van Kleef, 2012).

As anticipated, each Dark Tetrad trait correlated positively with the negative influence motive for gossiping. When controlling for the shared variance among dark traits, Machiavellianism, psychopathy, and sadism positively predicted this motive, whereas narcissism did not, providing partial support for Hypothesis 2. In previous work, only narcissism was positively related to both prestige and dominance status-striving (Davis & Vaillancourt, 2023). To obtain likeability and respect as well as greater social rank via intimidation, narcissists might transmit less negative gossip compared to those with the other Dark Tetrad characteristics. Nonetheless, narcissism is positively associated with indirect aggression, both concurrently and longitudinally (Farrell & Vaillancourt, 2021). This trait does appear to be less malevolent relative to other dark dimensions of personality (Furnham et al., 2013), and its predictive power might be statistically suppressed in the context of multivariate analyses. Our exploratory analysis supported this conclusion; psychopathy significantly suppressed narcissism in predicting the negative influence gossip motive. Similar suppression effects have been found in previous work (e.g., Davis et al., 2019b). This further speaks to the difficulty in interpreting the unique contributions of the Dark Tetrad traits when using multivariate techniques like multiple regression (Miller et al., 2019).

Information gathering and validation may perceivably be the most important gossip motive (Hartung et al., 2019). However, adults do seem highly inclined to believe in the veracity of gossip (Dores Cruz et al., 2021b). Partial support was provided for Hypothesis 3: Machiavellianism emerged as the only positive predictor of the information gathering and validation motive, whereas narcissism did not. Lyons and Hughes (2015) found that none of the Dark Triad dimensions predicted information gathering and validation. Studying information gathering and information validation as distinct motives, Hartung et al. (2019) found that narcissism positively predicted information gathering in a workplace context (but not a private context), as well as information validation in private and workplace contexts. Some have argued that higher levels of narcissism might predispose individuals to believe that gossip about them is likely to be positive (Hales et al., 2025), which could render them less likely to carefully vet gossiped information. In contrast, Machiavellianism has positively predicted gossip for information gathering in a private, but not a workplace, context (Hartung et al., 2019). Surprisingly, Machiavellianism did not predict information validation in the study by Hartung et al. (2019). Theoretically, Machiavellianism, more than the other traits within the Dark Tetrad, should logically be related to this motive. Cynicism is core to Machiavellianism (Blötner & Bergold, 2022; Christie & Geis, 1970), and distrust in others should engender more information validation and vetting gossip carefully. The same cannot be said for narcissism. Thus, our results, despite contrasting with those of some previous researchers (Hartung et al., 2019; Lyons & Hughes, 2015), are more aligned with theoretical perspectives on Machiavellianism.

In previous work, secondary psychopathy (i.e., impulsivity) and narcissism positively predicted the group protection gossip motive in a multiple regression model (Lyons & Hughes, 2015). Narcissism (but not psychopathy) has also been found to positively predict this motive in a workplace context (Hartung et al., 2019). In the present study, each Dark Tetrad trait correlated positively with group protection, but only Machiavellianism and psychopathy predicted this motive when accounting for the shared variance among the dimensions, thus providing partial support for Hypothesis 4. Given that psychopathy is linked to callousness and a lack of affective empathy (Hare et al., 1991; Shukla & Upadhyay, 2025), it may be surprising that this dark trait positively predicted the group protection gossip motive. Nonetheless, the appeal of groups typified by antisociality (e.g., gangs) often revolves around group protection, even if it places individuals at an increased risk of victimization (Melde et al., 2009). Moreover, secondary psychopathy (which resonates more with Dark Tetrad psychopathy) has been linked to fearless dominance, denoting boldness and reduced threat sensitivity (Lilienfeld et al., 2012). Those higher in psychopathy fiercely pursue dominance status using indirect aggression (Davis & Vaillancourt, 2023). Strict dominance hierarchies tend to characterize antisocial groups, which maintains group structure and reduces overall violence (Nakamura et al., 2020). This research suggests that it can be advantageous for those higher in psychopathy to protect in-group members in antisocial groups and organizations.

Narcissists are egoistic (Raskin & Terry, 1988) and value self-enhancement (Petrov et al., 2026), which theoretically conflicts with the notion that this trait should predict concerns over group protection. However, group protection does connect with the construct of collective narcissism, whereby individuals have an exaggerated and grandiose image of the social groups to which they belong (Golec de Zavala et al., 2019). It may then be collective, rather than individual, narcissism that is linked with gossiping for the sake of group protection. Future researchers could examine this possibility.

Machiavellians are expected to strategically oscillate between prosocial (e.g., cooperation) and antisocial (e.g., bullying) behaviour depending on situational demands to obtain desired resources, such as power and status (Davis & Vaillancourt, 2023; Hawley, 2006, 2014; Jones & Mueller, 2022). Thus, it is sensible for these individuals to have a more diverse range of motives underpinning their gossip. The same cannot be said for the other members of the Dark Tetrad. Theoretically, Machiavellians would be expected to ignore others and cut relational ties that afford no clear long-term benefit. However, as long as individual and group interests remain aligned, those higher in Machiavellianism may prioritize gossiping for the purpose of group protection.

In our exploratory analyses, we tested the possibility that psychopathy and sadism may have been redundant in predicting the group protection gossip motive. Some researchers (e.g., Jonason et al., 2017) have argued psychopathy can have this influence on sadism in predicting certain outcomes. However, there was insufficient evidence to support this conclusion. Psychopathy accounted for a significant proportion of the variance in predicting group protection above the influence of sadism, whereas sadism no longer predicted this outcome with the inclusion of psychopathy into the regression model.

### Limitations and Future Directions

The current research addresses a salient gap in the gossip literature and helps to clarify mixed findings with a relatively large sex-balanced sample of North American adults. Nonetheless, the current research has important limitations. When asked to self-report their gossip frequency, people may not have reliable insight into this behaviour (Foster, 2004). Alternatively, individuals may rate themselves as less likely to gossip because they are aware of the reputational damage associated with being perceived as a “gossip” (Farley, 2011; Hartung & Renner, 2013). However, those higher in certain “dark” personality traits, such as psychopathy and sadism, are likely less concerned with this potential reputational damage. Nonetheless, researchers would benefit from studying gossip more naturalistically or in experimental contexts that go beyond self-report instruments (e.g., Robbins & Karan, 2020).

Importantly, each Dark Tetrad dimension embodies lower-order facets (Buckels et al., 2013; Miller et al., 2019). In the literature on gossip, some researchers have studied these dark personality traits using different theoretical perspectives and operational definitions. For example, Lyons and Hughes (2015) studied primary (callous–unemotional) and secondary psychopathy (impulsive and antisocial) in relation to gossip motives. With the Short Dark Tetrad (SD4; Paulhus et al., 2020), psychopathy is understood and measured more in line with secondary psychopathy. Alternative measures could be employed to tap into the multidimensionality of each Dark Tetrad trait. For instance, grandiose and vulnerable narcissism could be examined using the Five Factor Narcissism Inventory (FFNI; Miller et al., 2013). Therefore, in future work it would be prudent to assess some of the different facets and operational definitions of the Dark Tetrad dimensions in relation to gossip frequency and motives.

The Gossip Frequency Scale used for the current study was adapted from the Tendency to Gossip Questionnaire (TGQ; Nevo et al., 1993). This adapted version of the scale demonstrated evidence of reliability (e.g., internal consistency) and validity (e.g., convergent validity) and provided participants with a definition of gossip to reduce interpretive ambiguity, but it has not been subjected to an in-depth psychometric analysis. Therefore, researchers should continue to examine the links between the Dark Tetrad and the newly adapted Gossip Frequency Scale to ensure its continued psychometric adequacy. This measure, however, does not permit an assessment of gossip valence (e.g., positive vs. negative gossip) or how gossip varies across contexts. In the future, researchers could use measures such as the Workplace Gossip Scale (Brady et al., 2017) to examine the links between the Dark Tetrad and positive and negative workplace gossip. Topics of gossip (e.g., rivals, friends, etc.) were also aggregated to calculate a total gossip frequency score in the current study. To better assess gossip toward specific targets, in the future, researchers could use vignettes describing specific scenarios (e.g., sibling rivalry; Hess & Hagen, 2021) and examine the associations between the Dark Tetrad and the use of positive and negative gossip.

Researchers also continue to examine the factor structure of the Motives to Gossip Questionnaire (Beersma & Van Kleef, 2012). Some have used a six-factor solution, where information gathering and information validation are measured separately, with the addition of a relationship-building factor (Hartung et al., 2019). Others find that a five-factor solution best fits the data with a combined information gathering and validation factor, as well as a venting emotions factor (Dores Cruz et al., 2019). Therefore, investigators could study the links between the Dark Tetrad and gossip motives using the Motives to Gossip Questionnaire-Revised to assess gossip motives more rigorously.

We also employed a variable-centered approach to examining the links between the Dark Tetrad with gossip frequency and gossip motives. Person-centered analytic techniques (e.g., latent profile analysis) allow for an examination of heterogenous groups regarding varying levels of personality dimensions and related behaviour (e.g., Farrell et al., 2025). These kinds of methods can complement the variable-centered analyses used in the current study. It would also be advantageous to use these analytic techniques with longitudinal data (e.g., Farrell & Vaillancourt, 2023).

## 5. Conclusions

Those expressing elevated levels of the Dark Tetrad traits, particularly psychopathy, are more inclined to exchange information that is negative, neutral, and positive to absent third-party others. Each dimension within the tetrad was positively associated with gossiping for information gathering and validation, social enjoyment, negative influence, and group protection. However, differences among the Dark Tetrad emerged when accounting for their shared variance, with Machiavellianism being the only dimension that positively predicted each gossip motive. This aligns with notions of Machiavellians as cynical, manipulative, and calculated strategists who employ a variety of prosocial and antisocial tactics to achieve their long-term goals (Hawley, 2006, 2014). Narcissism positively predicted social enjoyment, whereas psychopathy positively predicted the negative influence and group protection motives. Sadism also predicted transmitting gossip for the purpose of damaging the reputations of others. The current research builds on past empirical work regarding “dark” personality traits, gossip frequency, and gossip motives (e.g., Hartung et al., 2019; Lyons & Hughes, 2015; Uçan & Avci, 2023). It helps to gather further insight and clarity into the adaptive utility of gossiping, the diverse goals underpinning the behaviour, and the role of individual differences in personality that impact gossip frequency and motives.

## Figures and Tables

**Table 1:** Previous Research on the Dark Triad Dimensions with Gossip.

Study	Key Measures	Key Findings
Lyons and Hughes (2015)	Self-Reported Psychopathy Scale III (SRP-II; Paulhus et al., in press) Mach IV Scale (Christie & Geis, 1970) Narcissistic Personality Inventory (NPI; Raskin & Terry, 1988) Motives to Gossip Questionnaire (MGQ)	Dark Triad traits (+) correlated with negative influence motive Narcissism (+) correlated with social enjoyment and group protection motives Primary psychopathy and narcissism (+) linked to social enjoyment motive Secondary psychopathy and narcissism (+) linked to group protection
Hartung et al. (2019)	Motives to Gossip Questionnaire-Revised (MGQ-R; Hartung et al., 2019) The Dirty Dozen Scale (Jonason & Webster, 2010)	Dark Triad traits (+) correlated with negative influence motive Narcissism (+) predicted information validation and relationship-building motives in workplace and private settings Machiavellianism (+) predicted information gathering in workplace and private settings
Stiff (2019)	Short Dark Triad (SD3; Jones & Paulhus, 2014) Attitudes toward Gossip Scale (Litman & Pezzo, 2005)	Machiavellianism (+) predicted positive attitudes toward gossiping (i.e., endorsing gossip)
Uçan and Avci (2023)	The Dirty Dozen Scale (Jonason & Webster, 2010) Organizational Gossip Scale (Han & Dağli, 2018)	Dark Triad traits correlated (+) with information gathering and relationship-building gossip motives in a workplace setting
Wantante (2024)	Adapted version of The Dirty Dozen Scale (Jonason & Webster, 2010) The Positive and Negative Gossip About Managers Scale (Ellwardt et al., 2012)	Perceived Dark Triad traits in supervisors (+) correlated with negative gossip about managers
Hales et al. (2025)	Four gossip scenario vignettes (negative, ambiguous, no gossip, positive) Single-Item Narcissism Scale (SINS; Konrath et al., 2014) NPI (Raskin & Terry, 1988)	Narcissism (+) correlated with openness to being gossiped about across scenarios

**Table 2:** Descriptive Statistics and Bivariate Correlations.

	1.	2.	3.	4.	5.	6.	7.	8.	9.
1. Narcissism	----								
2. Machiavellianism	0.44	----							
3. Psychopathy	0.65	0.41	----						
4. Sadism	0.54	0.50	0.74	----					
5. Gossip Frequency	0.56	0.43	0.66	0.57	----				
6. Info. Gather. & Valid.	0.20	0.34	0.20	0.20	0.41	----			
7. Social Enjoyment	0.29	0.37	0.27	0.25	0.47	0.53	----		
8. Negative Influence	0.34	0.39	0.53	0.49	0.52	0.48	0.32	----	
9. Group Protection	0.32	0.28	0.35	0.28	0.43	0.57	0.34	0.48	----
*N*	516	516	516	516	516	516	516	516	516
Minimum	1.00	1.00	1.00	1.00	1.00	1.00	1.00	1.00	1.00
Maximum	5.00	5.00	4.86	4.86	4.92	7.44	7.20	7.40	8.00
*M*	2.88	3.39	2.17	2.54	2.51	4.72	4.32	3.20	4.11
*SD*	0.93	0.77	1.01	1.00	0.82	1.43	1.52	1.51	1.50

*Note*. All bivariate correlations significant at *p* < 0.001 (two-tailed) with pairwise deletion.

**Table 3:** Multiple Regression Analyses for Dark Tetrad Predicting Information Gathering & Validation and Social Enjoyment Motives.

	Gossip Motives					
	Info. Gathering & Validation			Social Enjoyment		
	*b*	β	*p*	*b*	β	*p*
Step 1						
Age	−0.00	−0.02	0.671	−0.02	**−0.16**	<0.001
Sex	−0.20	−0.07	0.129	−0.19	−0.06	0.163
*R* ^2^		0.01			0.03	
Step 2						
Narcissism	0.02	0.01	0.811	0.20	**0.12**	0.026
Mach.	0.59	**0.32**	<0.001	0.54	**0.28**	<0.001
Psych.	0.09	0.06	0.378	0.09	0.06	0.382
Sadism	0.05	0.04	0.591	0.00	0.00	0.981
*R* ^2^		0.14			0.16	

*Note*: *b* = unstandardized regression coefficient; β = standardized regression coefficient; statistically significant (*p* < 0.05) β values bolded; *R*^2^ = coefficient of determination; sex coded: 1 = female, 2 = male; pairwise deletion used.

**Table 4:** Multiple Regression Analyses for Dark Tetrad Predicting Negative Influence and Group Protection Motives.

	Gossip Motives					
	Negative Influence			Group Protection		
	*b*	β	*p*	*b*	β	*p*
*Step 1*						
Age	−0.03	**−0.21**	<0.001	−0.01	−0.05	0.231
Sex	−0.20	−0.07	0.137	−0.21	−0.07	0.116
*R* ^2^		0.05			0.01	
*Step 2*						
Narcissism	−0.12	−0.08	0.128	0.17	0.10	0.062
Mach.	0.34	**0** **.17**	<0.001	0.30	**0** **.16**	0.001
Psych.	0.54	**0** **.36**	<0.001	0.35	**0** **.24**	<0.001
Sadism	0.30	**0** **.20**	0.001	0.01	0.01	0.926
*R* ^2^		0.34			0.16	

*Note*: *b* = unstandardized regression coefficient; β *=* standardized regression coefficient; statistically significant (*p* < 0.05) β values bolded; *R*^2^ = coefficient of determination; sex coded: 1 = female, 2 = male; pairwise deletion used.

**Table 5:** Suppressor and Redundancy Regression Analyses.

*Y* = Negative Influence Gossip Motive					
*X*_1_ = Narcissism			*X*_1_ = Psychopathy		
β alone	β with Psychopathy	*R*^2^ Δ	β alone	β with Narcissism	*R*^2^ Δ
0.31 ***	−0.00	0.15 ***	0.51 ***	0.51 ***	0.00
*Y* = Group Protection Gossip Motive					
*X*_1_ = Sadism			*X*_1_ = Psychopathy		
β alone	β with Psychopathy	*R*^2^ Δ	β alone	β with Sadism	*R*^2^ Δ
0.32 ***	0.09	0.04 ***	0.37 ***	0.30 ***	0.00

*Note*. Age and sex statistically controlled for at Step 1; results significant at *** *p* < 0.001.

## Data Availability

Data are available upon request of the corresponding author. The data are not publicly available due to privacy and ethical restrictions.

## Note

1 Exploratory analyses indicated that sex did not moderate any of the links between the Dark Tetrad and gossip frequency or gossip motives. Effect coding was used for sex (−0.50 = female, 0.50 = male) and each predictor variable was mean-centered in the multiple regression analyses.

## Supplementary Materials

The following supporting information can be downloaded at: https://www.mdpi.com/article/10.3390/bs16091556/s1; Gossip Frequency Scale.

## Abbreviations

The following abbreviations are used in this manuscript:

MTurk	Amazon Mechanical Turk
SD4	Short Dark Tetrad 4
HIT	Human intelligence task
TGQ	Tendency to Gossip Scale
MGQ	Motives to Gossip Questionnaire
