# Supplementary material for: The Dark Tetrad Traits Differentially Predict Gossip Frequency and Motives
Source: Behav Sci (Basel). 2026 Sep 2;16(9):1556. doi: 10.3390/bs16091556 (PMC13603136; doi:10.3390/bs16091556)
Supplement: Supplementary file 1 [file behavsci-16-01556-s001.zip › behavsci-4443626-supplementary.pdf]

Supplementary Materials

Gossip Frequency Scale

Instructions: Gossip involves sharing information with other people about someone who is not currently present. Gossip can be positive (e.g., talking about someone else's achievements), negative (e.g., making fun of someone behind their back), or neutral. Gossip can take place in a face-to-face conversation, on the phone, through email, text messaging, or on social media.

Using the scale provided, please indicate how often you gossip about:

1	2	3	4	5	0
Never	Rarely	Sometimes	Often	Always	Skip

1. Other people's achievements at school (e.g., grades) or work (e.g., salaries)
2. Other people's physical appearance (e.g., body size, clothing, makeup, etc.)
3. Other people's romantic relationships (e.g., compatibility of couples, other people's love affairs, and/or who is dating who)
4. My friends' and/or other people's friendships
5. Bosses, supervisors, and/or professors
6. Family members
7. Rivals, competitors, and/or enemies
8. Actresses and/or actors
9. Professional athletes
10. Politicians
11. Popular scientists
12. Authors of books, magazines, and/or newspaper articles
13. Bloggers, vloggers, YouTube personalities, and/or podcasters
